# Direct Electrochemistry and Electrocatalysis of Hemoglobin at Mesoporous Carbon Modified Electrode

**DOI:** 10.3390/s100201279

**Published:** 2010-02-03

**Authors:** Supeng Pei, Song Qu, Yongming Zhang

**Affiliations:** 1 School of Chemistry and Chemical Engineering, Shanghai Jiao Tong University, Shanghai 200240, China; E-Mail: peisupeng@sjtu.edu.cn; 2 College of Science, University of Shanghai for Science and Technology, Shanghai 200093, China

**Keywords:** hemoglobin, mesoporous carbon, direct electron transfer

## Abstract

The novel highly ordered mesoporous carbon (known as FDU-15), prepared by the organic-organic self-assembly method was been used for first time for the immobilization of hemoglobin (Hb) and its bioelectrochemical properties were studied. The resulting Hb/FDU-15 film provided a favorable microenvironment for Hb to perform direct electron transfers at the electrode. The immobilized Hb also displayed its good electrocatalytic activity for the reduction of hydrogen peroxide. The results demonstrate that mesoporous carbon FDU-15 can improve the Hb loading with retention of its bioactivity and greatly promote the direct electron transfer, which can be attributed to its high specific surface area, uniform ordered porous structure, suitable pore size and biocompatibility. Our present study may provide an alternative way for the construction of nanostructure biofunctional surfaces and pave the way for its application to biosensors.

## 1. Introduction

The realization of direct electron transfer between the native redox protein and the underlying electrode is significantly important because it not only provides models for studying the mechanism of biological electron transport, but also enables construction of mediator-free biosensors and bioreactors [1,2]. Hemoglobin (Hb) is a heme protein which can store and transport oxygen in the blood in vertebrates. Because of its commercial availability and well-documented structure, Hb is an ideal model molecule for the study of direct electron transfer reactions and electrocatalysis of heme proteins. However, because of its large structure, it is difficult for Hb to directly exchange electrons with an electrode surface. Therefore, developing suitable materials and methods for effective Hb immobilization on electrode surface is important for achieving their direct electrochemical reactions and retaining their bioactivities. During the past two decades, great efforts have been made to increase the electron transfer kinetics of Hb [3–10].

Mesoporous materials offer new possibilities for immobilization of proteins because they are porous materials with extremely high surface areas and uniform pores [6,11–13]. Mesoporous carbon materials with ordered pore structure, high pore volume, high specific surface area, and tunable pore diameters have been widely investigated in various areas such as catalyst supports, electrode materials, molecular separation and so on [14–17]. In particular, the order mesoporous carbon materials also have been widely applied in electrochemical biosensors [18–20].

The ordered mesoporous carbon materials have been usually prepared by the nanocasting method using hard-templates [21]. Recently, a direct synthesis of ordered mesoporous carbons through an organic–organic self-assembly method was applied to prepare some ordered mesoporous carbon materials [22–24]. The pore wall structures of these carbons are different from those of mesoporous carbon prepared from nanocasting method using hard templates. For example, a two dimensional (2-D) hexagonal mesostructured carbon FDU-15 prepared by Zhao *et al.* possess continuous and open frameworks with ultrahigh thermal stability in inert atmospheres, which arises from the covalently bonded construction, amorphous carbon components and thick pore walls [22]. These carbonaceous materials possess high surface area, large pore volumes and uniform pore structure. Considering their especial properties, the ordered mesostructured FDU-15 carbons could be used as attractive materials for protein immobilization.

In this report, the highly ordered mesoporous carbon FDU-15 was used for Hb immobilization and then its bioelectrochemical properties were studied. The direct electron transfer of Hb was observed on the Hb/FDU-15 modified electrode. The resulting film provided a desirable microenvironment to retain the bioactivity of Hb. The electrocatalytic reduction of H_2_O_2_ at the modified electrode was also investigated. It represents a general method for the construction of biosensor and can be applied to other biosystems.

## 2. Experimental

### 2.1. Reagents

Pluronic F127 triblock poly(ethyleneoxide)-b-poly(propylene oxide)-b-poly(ethylene oxide) copolymer (MW = 12,600, EO106PO70EO106) was purchased from Acros Corp. Hb (MW 66,000, from bovine blood) was purchased from Shanghai Biochemical Reagent and used without further purification. Other chemicals were purchased from Shanghai Chemical Company. All these chemicals were used of analytical grade or higher and used as received. All the solutions were prepared with doubly distilled water.

### 2.2. Syntheses of mesoporous carbon FDU-15

Mesoporous carbon FDU-15 with 2-D hexagonal structures was synthesized through an organic–organic self-assembly method, according to the literature [22]. Briefly, 1.0 g of F127 block copolymer was dissolved in 20.0 g of ethanol. Then 5.0 g of the resol precursors in ethanol solution containing 0.60 g of phenol and 0.45 g of formaldehyde were added under stirring. The homogeneous solution was poured into several dishes to evaporate ethanol at room temperature for 8 h and then heated at 100 °C for 24 h. The as-prepared products were calcined in a tubular furnace under a high purity N_2_ atmosphere at 900 °C for 3 h.

### 2.3. Electrode modification

One mg of mesoporous carbon FDU-15 was dispersed in 1 mL dimethylformamide (DMF) with the aid of ultrasonic agitation to give a 1 mg mL^−1^ black suspension. 5 mg mL^−1^ Hb solution was prepared by dissolving Hb in 0.10 M phosphate buffer solution (PBS) at pH 7.0. Prior to modification, the bare glassy carbon (GC) electrode was polished with 0.05 μm alumina slurry and then ultrasonically cleaned in ethanol and water, followed by thoroughly rinsing with water. Then 10 μL of the suspension was spread on the pretreated GCE surface and allowed to dry under an infrared lamp. The mesoporous carbon FDU-15 electrode obtained was thoroughly rinsed with water and immersed in a 5 mg mL^−1^ Hb solution for 24 h to give the Hb/FDU-15 modified electrode.

### 2.4. Apparatus and measurements

Transmission electron microscopy (TEM) micrographs of samples were taken using a JEM-2011 electron microscope (JEOL, Japan), with an accelerating voltage of 200 kV. Small-angle X-ray scattering (SAXS) measurements were taken on a Nanostar U smallangle X-ray scattering system (Bruker, Germany) using Cu Ka radiation (40 mV, 35 mA). N_2_ adsorption was measured using a Micromeritics Tristar 3000 automatic physisorption instrument at 77 K. The specific surface area was determined by the BET method. The BJH model was used to determine the pore size distribution.The UV-Vis spectras were measured with a JASCO UV550 UV–Vis absorption spectrometer.

Electrochemical experiments were performed on a CHI 660 electrochemical workstation (CHI, USA) with conventional three-electrode system. The working electrode was a modified GC electrode. A saturated calomel electrode (SCE) and a platinum electrode were used as the reference and the auxiliary electrode, respectively. A 0.10 M PBS was used as the supporting electrolyte. All experimental solutions were deoxygenated by purging pure N_2_ into the solution for about 15 min, and N_2_ gas was kept flowing over the solution during the electrochemical measurements.

## 3. Results and Discussion

### 3.1. Characterization of FDU-15

**Figure 1.**
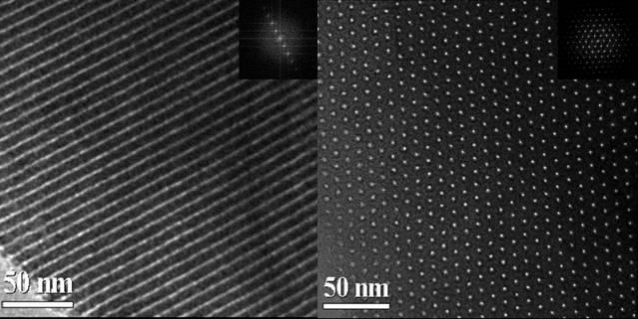
TEM images of the mesoporous carbon FDU-15.

Transmission electron microscopy (TEM) images of mesoporous carbon FDU-15 (Figure 1) show well ordered stripe-like and hexagonally arranged patterns that are similar to mesoporous silica SBA-15, further confirming a 2D ordered hexagonal mesostructure [25]. The wall thickness is estimated to be 4.0 nm.

**Figure 2.**
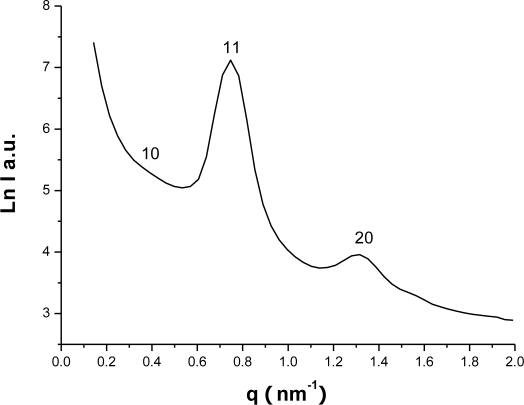
SASX patterns of the mesoporous carbon FDU-15.

**Figure 3.**
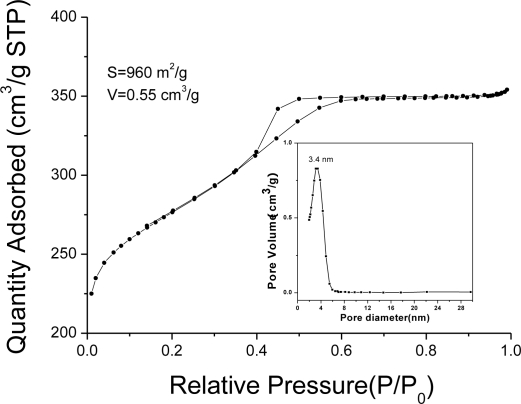
N_2_ adsorption curves of the mesoporous carbon FDU-15.

SAXS patterns (Figure 2) show that the two-dimensional (2D) hexagonal structure (p6 mm) with three distinct reflection peaks indexed to10, 11 and 20 respectively. The N_2_ sorption isotherms (shown as circles in Figure 3) of the mesoporous carbon FDU-15 yield a type-IV curve with a sharp capillary condensation step at *P*/*P*_0_ = 0.4 – 0.6 and an H_1_-type hysteresis loop which is typical of large-pore mesoporous materials with cylindrical channels, according to the International Union of Pure and Applied Chemistry(IUPAC) nomenclature[26]. These results suggest that mesoporous carbon FDU-15 has uniform cylindrical mesoporous channels. The BET surface area and the total pore volume are calculated to be 960 m^2^ g^−1^ and 0.55 cm^3^ g^−1^, respectively. The pore diameter is about 3.4 nm with a narrow distribution.

### 3.2. Direct electrochemistry of Hb on Hb/FDU-15 electrode

**Figure 4.**
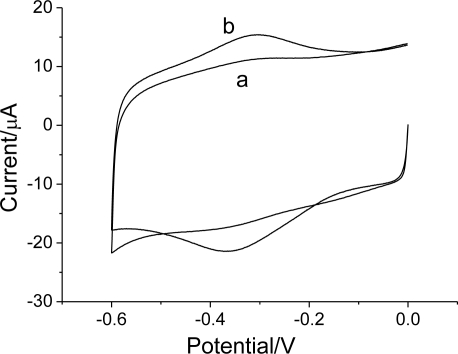
Cyclic voltammograms of (a) FDU-15 and (b) Hb/FDU-15 electrodes in pH 7.0 PBS; scan rate: 100 mV s^−1^.

Cyclic voltammetry was employed to investigate the Hb/FDU-15 film over the potential range from 0 to −0.6 V. Figure 4 shows the cyclic voltammograms of mesoporous carbon FDU-15/GC and Hb/FDU-15 electrodes at a scan rate of 100 mV s^−1^, respectively. As compared to GC electrode (not shown), the electrochemical response current at the FDU-15/GC modified electrode is much larger. It can be attributed to the electrode surface area of FDU-15/GC modified electrode is significant increment. The similar phenomenon could be also observed at CNTs modified electrode [27].

No peak was observed at mesoporous carbon FDU-15 (curve a) modified electrode, which showed mesoporous carbon FDU-15 was electroinactive within the potential window. However, a pair of well-defined, nearly symmetrical redox peaks is observed at the Hb/FDU-15 electrode (curve b). The anodic peak potential (*E_pa_*) and cathodic peak potential (*E_pc_*) are located at −0.304 and −0.357 V, respectively. The formal potential (*E*^0’^), estimated as the average of *E_pa_* and *E_pc_*, is −0.33 V. The peak-to-peak separation (Δ*E_p_*) of 53 mV is observed, which indicates a fast direct electron transfer of Hb in the film. This is in accordance with the characteristic of Fe(III)/Fe(II) redox couples of heme proteins.

The direct electrochemistry of Hb indicates that the ordered mesoporous carbon FDU-15 film has good affinity for proteins. Hb has been adsorbed or entrapped by the film for the film porosity. Moreover, the film could greatly facilitate the electrode reaction kinetics of Hb, and provide a favorable microenvironment for electron transfer between Hb and electrode.

**Figure 5.**
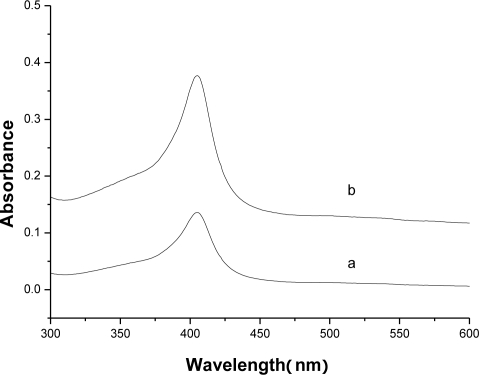
UV-Vis spectroscopy of (a)Hb/FDU-15 film and (b)Hb in pH 7.0 PBS.

Figure 5 shows the UV-Vis spectrum of the Hb/FDU-15 film. It can be observed that the Soret band of the native Hb is located at 403 nm (Figure 5b). After Hb is immobilized, the Soret band is still appeared at 403 nm and the wavelength has no obvious shifted (Figure 5a). The absorption peak at 403 nm is the characteristic Soret-band absorbance of the heme of Hb [28], indicating that the FDU-15 can provide a microenvironment for Hb to retain its native structure.

**Figure 6.**
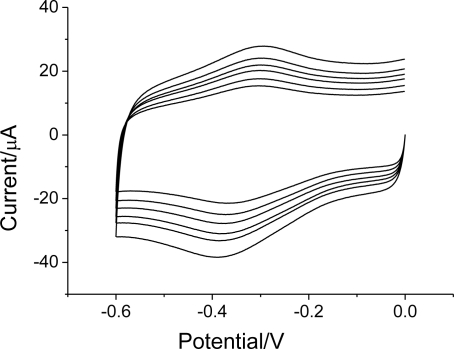
Cyclic voltammograms of Hb/FDU-15 modified electrode in pH 7.0 PBS at scan rate of 100, 125, 150, 175, 200 and 250 mV s^−1^.

The influence of scan rate on the response of immobilized Hb in the film was investigated (Figure 6). The linear regression equations of peak current with scan rate is: *I_pa_*(uA) = 0.038 + 0.033 v (mV s^−1^) *I_pc_*(uA) = 0.012 + 0.037 v (mV s^−1^).

Both the anodic and cathodic peak currents increase linearly with the scan rates (from 100 mV s^−1^ to 250 mV s^−1^), indicating that the reaction is a surface controlled electrochemical process. According to the slope of the Ip–v curve of *I_p_* = n^2^F^2^vΓA/4RT [29], the average surface coverage (Γ) of Hb was estimated to be 7.17 × 10^−11^ mol cm^−2^, which is higher than that of the theoretical monolayer coverage(1.89 × 10^−11^ mol cm^−2^) [30]. The high loading of Hb is attributed to the porous structure of the ordered mesoporous carbon FDU-15 with its large specific surface area.

When the scan rates increase, the *E_pa_* and *E_pc_* shift slightly to the positive and the negative directions, respectively. The electrode reaction becomes irreversible at higher scan rates. Using Laviron’s equation [31,32], the heterogeneous electron transfer rate constant k of Hb can be calculated as 1.8 s^−1^.

**Figure 7.**
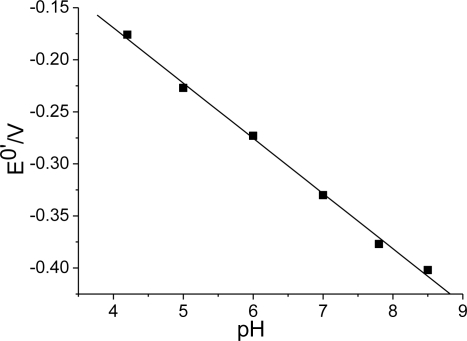
The dependence of *E*^0’^ of the Hb/FDU-15 electrode on the solution pH.

The effect of pH on the peak potential of Hb immobilized in the film was investigated in different buffer solutions. The dependence of *E*^0’^ on the pH at the Hb/FDU-15 electrode is shown in Figure 7. It can be observed that the peak potential is obviously dependent on the pH value in the range of 4.0–9.0. The *E*^0’^ of Hb shifts linearly to the negative direction with increasing pH value with a slope of −53 mV pH^−1^. The slope of the *E*^0’^–pH plots are consistent with the mechanism in which a proton and an electron are transferred per heme group in the electrode reaction.

### 3.3. Electrocatalysis of H_2_O_2_ on Hb/FDU-15 electrode

**Figure 8.**
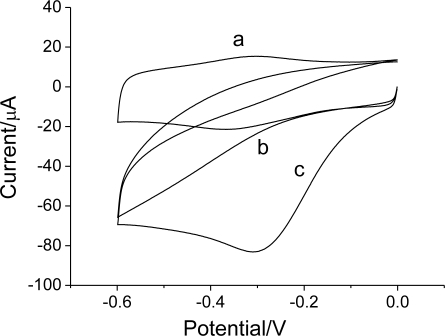
Cyclic voltammograms of the Hb/FDU-15 electrode in 0.1 M PBS (pH 7.0) (curve a); the FDU-15 electrode (b) and the Hb/FDU-15 electrode (c) in the presence of 0.5 mM H_2_O_2_. Scan rate: 100 mV s^−1^.

Hb has the ability to catalyze the reduction of H_2_O_2_. Figure 8 shows the cyclic voltammograms of the FDU-15 (curve b) and Hb/FDU-15 (curve c) electrode in 0.1 M PBS (pH 7.0) in the presence of H_2_O_2_.

As for Hb/FDU-15 electrode, in the absence of H_2_O_2_, a pair of the redox peaks of Hb is observed (curve a). However, in the presence of H_2_O_2_, the voltammetric behavior changes drastically. A large cathodic current for the reduction of H_2_O_2_ appears while the anodic peak decreases till disappears completely. Compared with that, at FDU-15 electrode, no voltammatry response is observed in the same condition. The results indicate that H_2_O_2_ exhibits excellent reactivity in the electrocatalysis on the Hb/FDU-15 electrode. The porous structure of mesoporous carbon FDU-15 can provide sufficient space to immobilize Hb and give the immobilized Hb a suitable micro-environment to keep their biological activity.

**Figure 9.**
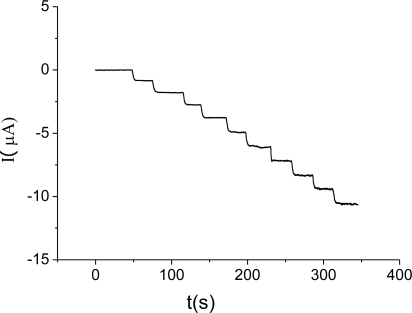
Typical steady-state current responses of the Hb/FDU-15 electrode on successive addition of 10 μM H_2_O_2_. Applied potential: −0.3 V.

An attractive feature of the Hb/FDU-15 electrode is the highly stable amperometric response to H_2_O_2_. Figure 9 shows the typical steady-state current responses of the modified electrode by the successive addition of 10 μM H_2_O_2_ at −0.3 V.

The current responses to the addition of H_2_O_2_ quickly and sensitively and reaches a steady state in 4 s. Such a fast response can be attributed to the fast diffusion process and a high activity of the Hb in this system. The current is linear with the H_2_O_2_ concentration from 2 × 10^−6^ M to 3 × 10^−4^ M with the detection limit of 8 × 10^−7^ M (S/N = 3). Compared with other CNTs-based H_2_O_2_ biosensors [33,34], the linear range of our proposed biosensor is broader by about one order of magnitude of H_2_O_2_ concentration with a markedly lower detection limit. That indicates the hydrogen peroxide biosensor based on Hb/FDU-15 would exhibit more advantageous analytical performance. This improved analytical performance is because FDU-15 has the ability to promote the electron transfer between proteins and electrode.

When the concentration of H_2_O_2_ is higher than 5 × 10^−4^ M, a response plateau is observed, showing the characteristics of the Michaelis–Menten kinetic mechanism. The apparent Michaelis–Menten constant (K_m_), which is an indication of the enzyme–substrate kinetics, can be calculated using the Lineweaver–Burk equation(1) [35]:

(1) $$\frac{1}{i}=\frac{1}{I_{\text{max}}}+\frac{K_{m}^{\mathrm{app}}}{I_{\text{max}}}\cdot \frac{1}{C_{H_{2}O_{2}}}$$

Accordingly, the K_m_ value for the Hb/FDU-15 modified electrode is estimated to be 1.38 mM. This value is smaller than that of Hb/CNTs modified electrode [36]. The low value of K_m_ indicates that Hb immobilized in FDU-15 films retains its bioactivity and has a high biological affinity to H_2_O_2_ with a low diffusion barrier.

The repeatability and stability of the proposed electrode were studied. The relative standard deviation (RSD) is 3.6% for ten successive measurements of 10 μM H_2_O_2_, showing the proposed electrode possesses a good reproducibility. On the other hand, the storage stability of the proposed electrode was also studied. The response current of the electrode decreased to 90% after stored 30 days at 4 °C in a refrigerator. The good long-term stability demonstrated that the FDU-15 film was suitable matrix for immobilization of Hb to retain its activity and prevent it from leaking out of the film.

## 4. Conclusions

An application of mesoporous carbon FDU-15 in bioelectrochemical research has been studied. Mesoporous carbon FDU-15 possesses high specific surface, ordered pore structure, high pore volume. Thus mesoporous carbon FDU-15 could be used as an attractive materials for protein immobilization. Hb immobilized in the Hb/FDU-15 film has performed direct electrochemistry and retained high electrocatalytic efficiency toward H_2_O_2_. The Hb/FDU-15 electrode exhibited good analytical performance feuatures, such as a wide determination range and low detection limit for H_2_O_2_ determination. Thus, mesoporous carbon FDU-15 as support for redox protein immobilization has potential applications in the fabrication of third-generation biosensors.
